# Small Study Effects in Diagnostic Imaging Accuracy: A Meta-Analysis

**DOI:** 10.1001/jamanetworkopen.2022.28776

**Published:** 2022-08-25

**Authors:** Lucy Lu, Qi Sheng Phua, Stephen Bacchi, Rudy Goh, Aashray K. Gupta, Joshua G. Kovoor, Christopher D. Ovenden, Minh-Son To

**Affiliations:** 1 College of Medicine and Public Health, Flinders University, Bedford Park, Australia; 2 Department of Neurology, Royal Adelaide Hospital, Adelaide, Australia; 3 Faculty of Health and Medical Sciences, The University of Adelaide, Adelaide, Australia; 4 Department of Neurology, Lyell McEwin Hospital, Elizabeth Vale, Australia; 5 Department of Cardiothoracic Surgery, Gold Coast University Hospital, Southport, Australia; 6 Department of Surgery, The Queen Elizabeth Hospital, Woodville South, Australia; 7 Department of Neurosurgery, Royal Adelaide Hospital, Adelaide, Australia; 8 South Australia Medical Imaging, Flinders Medical Centre, Bedford Park, Australia

## Abstract

**Importance:**

Small study effects are the phenomena that studies with smaller sample sizes tend to report larger and more favorable effect estimates than studies with larger sample sizes.

**Objective:**

To evaluate the presence and extent of small study effects in diagnostic imaging accuracy meta-analyses.

**Data Sources:**

A search was conducted in the PubMed database for diagnostic imaging accuracy meta-analyses published between 2010 and 2019.

**Study Selection:**

Meta-analyses with 10 or more studies of medical imaging diagnostic accuracy, assessing a single imaging modality, and providing 2 × 2 contingency data were included. Studies that did not assess diagnostic accuracy of medical imaging techniques, compared 2 or more imaging modalities or different methods of 1 imaging modality, were cost analyses, used predictive or prognostic tests, did not provide individual patient data, or were network meta-analyses were excluded.

**Data Extraction and Synthesis:**

Data extraction was performed in accordance with the PRISMA guidelines.

**Main Outcomes and Measures:**

The diagnostic odds ratio (DOR) was calculated for each primary study using 2 × 2 contingency data. Regression analysis was used to examine the association between effect size estimate and precision across meta-analyses.

**Results:**

A total of 31 meta-analyses involving 668 primary studies and 80 206 patients were included. Fixed effects analysis produced a regression coefficient for the natural log of DOR against the SE of the natural log of DOR of 2.19 (95% CI, 1.49-2.90; P < .001), with computed tomography as the reference modality. Interaction test for modality and SE of the natural log of DOR did not depend on modality (Wald statistic *P* = .50). Taken together, this analysis found an inverse association between effect size estimate and precision that was independent of imaging modality. Of 26 meta-analyses that formally assessed for publication bias using funnel plots and statistical tests for funnel plot asymmetry, 21 found no evidence for such bias.

**Conclusions and Relevance:**

This meta-analysis found evidence of widespread prevalence of small study effects in the diagnostic imaging accuracy literature. One likely contributor to the observed effects is publication bias, which can undermine the results of many meta-analyses. Conventional methods for detecting funnel plot asymmetry conducted by included studies appeared to underestimate the presence of small study effects. Further studies are required to elucidate the various factors that contribute to small study effects.

## Key Points

**Question:**

What are the prevalence and extent of small study effects in the diagnostic imaging literature?

**Findings:**

This meta-analysis of diagnostic performance data pooled from 31 diagnostic imaging accuracy meta-analyses including 668 primary studies found significant evidence for small study effects. Subgroup analysis by imaging modality revealed similar trends throughout all examined modalities (computed tomography, magnetic resonance imaging, positron emission tomography, ultrasonography).

**Meaning:**

These findings suggest small study effects are widely underestimated at the level of individual meta-analyses when using conventional methods, including visual assessment of funnel plots and Egger test.

## Teaser

This meta-analysis evaluates the presence and extent of small study effects in meta-analyses assessing diagnostic imaging accuracy.

## Introduction

Small study effects are the phenomena in which smaller studies have a tendency to show greater effect sizes than larger studies.^1^ Potential causes of small study effects are largely categorized into bias, heterogeneity, and pure coincidence.^2^ A well-known explanation for small study effects is publication bias,^3,4^ in which manuscripts that have statistically significant or favorable results are more likely to be published.^5^ This can contribute to small study effects, since smaller studies are more prone to publication bias.^6^ Another form of bias that can manifest as small study effects is outcome reporting bias, which is the selective reporting of only the more favorable outcomes.^2^

Small study effects caused by bias can impact meta-analyses, the criterion standard of evidence synthesis, by increasing the estimated pooled effect sizes.^4,7^ While meta-analysis is essential for summarizing evidence, the validity of a meta-analysis depends on the underlying data which, in turn, is influenced by multiple factors, such as methodological quality of the primary studies and presence of bias. Publication bias is not easily disentangled from other causes of small study effects, and detecting publication bias by retrieving unpublished data is typically not feasible.^8^ Thus, the commonly used methods to assess for publication bias, such as funnel plots, Egger test,^9^ and Deek test,^10^ are in fact tests to detect small study effects as a proxy of publication bias.^8^ Guidelines and checklists, such as the Preferred Reporting Items of Systematic Reviews and Meta-Analyses (PRISMA), have been developed to improve transparency and minimize bias.^5,11^ Although adherence to the PRISMA statement can improve the quality of a systematic review or meta-analysis, it cannot account for biases that are inherently present in the literature reviewed.^12^

Publication bias and small study effects have been well characterized among therapeutic and interventional studies.^13,14,15^ However, these phenomena are less understood in diagnostic accuracy research, and limited previous studies have produced varied results.^16,17^ There are fundamental differences between studies of diagnostic accuracy and interventional studies. In particular, most diagnostic accuracy studies do not have a predefined study hypothesis, while most randomized clinical trials of interventions test a prespecified hypothesis against the null hypothesis (of no difference in effect between participant groups).^18^ In diagnostic imaging accuracy studies, the accuracy of an imaging technique in differentiating whether a condition is present is determined through comparison with a reference standard. Such reference standards include an alternative imaging modality, histopathological examination, clinical diagnosis, or diagnosis on surgical exploration. Primary outcomes from diagnostic imaging studies include sensitivity, specificity, positive predictive value, and diagnostic odds ratio (DOR) measures.^19^ Given these differences from interventional studies, it is unclear how publication bias and small study effects impact diagnostic imaging research. Therefore, this study aimed to assess for the presence and magnitude of small study effects in diagnostic imaging accuracy meta-analyses.

## Methods

This meta-analysis did not require institutional board review. We did not register our meta-analysis. This review follows the PRISMA reporting guideline.^5^

### Search Strategy

The Journal Citation Reports^20^ of 2019 was used to identify the 30 journals with highest Impact Factor in the radiology, nuclear medicine, and medical imaging category. On December 26, 2020, we searched the PubMed database for meta-analyses on medical imaging accuracy studies published in those journals between January 1, 2010, and December 31, 2019. This date range was chosen to identify meta-analyses published after the PRISMA reporting guidelines were made available in 2009; however, primary studies published prior to this were not excluded. The search string is presented in the eAppendix in the Supplement.

### Eligibility Criteria

The inclusion criteria were meta-analyses that included 10 or more studies of medical imaging diagnostic accuracy, compared a single imaging modality against a reference standard (which may include imaging), and provided 2 × 2 contingency data (true positive [TP], true negative [TN]; false positive [FP]; false negative [FN]) for all included studies. The exclusion criteria were reviews without meta-analysis, studies that did not assess diagnostic accuracy of medical imaging techniques, studies that compared 2 or more imaging modalities or different methods of 1 imaging modality against a reference standard, cost analyses, analyses of predictive or prognostic tests, studies of individual patient data or network meta-analyses, and studies that did not provide 2 × 2 contingency data. Meta-analyses with fewer than 10 studies were also excluded, since existing tests for funnel plot asymmetry are underpowered when there are fewer than 10 studies in the meta-analysis.^2^ Meta-analyses that had overlapping data sets were identified, and only the most recent meta-analysis among those with overlapping data sets was included. Two of us (L.L. and Q.S.P.) independently conducted the screening, and any discrepancies were discussed with a third reviewer (M.-S.T.).

### Data Extraction

The following data were extracted from eligible meta-analyses: lead author of the study, year of publication, number of component primary studies, target condition, and imaging modality under evaluation. Imaging modality was classified as computed tomography (CT), magnetic resonance imaging (MRI), positron emission tomography (PET), ultrasonography, or other. Data extracted on primary studies within each meta-analysis were as follows: lead author of the study, year of publication, and TP, TN, FP, and FN data from 2 × 2 contingency tables. Studies within included meta-analyses that lacked complete 2 × 2 contingency data were individually removed prior to data analysis, and the meta-analysis was removed if the removal of primary studies with incomplete 2 × 2 contingency data resulted in fewer than 10 primary studies.

Additionally, the methods for assessment of publication bias used by the included meta-analyses and the results of the assessment were extracted. Specifically, we extracted whether the analysis used funnel plots, statistical tests, or both, and whether these tests found evidence for publication bias.

### Statistical Analysis

Data from the 2 × 2 contingency tables were used to calculate the sample size, number of participants with disease, number of participants without disease, and DOR. For the purposes of this study, DOR was used as the single value to represent diagnostic test accuracy to allow pooling and comparison of results from various meta-analyses. The use of a univariate measure, such as DOR, has also been suggested to reduce the risk of heterogeneity caused by different thresholds for diagnosis resulting in varying sensitivity and specificity, which may exaggerate or mask publication bias. Out of a number of univariate measures, the natural logarithm of DOR (ln[DOR]), specifically has been suggested to perform better in detecting publication bias.^21^ Per the Haldane-Anscombe correction,^22,23,24^ an adjustment of adding 0.5 was made to all values to correct for any entries of zero that would otherwise have resulted in an undefined DOR.

A composite funnel plot was first constructed by plotting the effect size against precision (ln[DOR] against SE) of all primary studies for visual assessment of asymmetry. Then, the *regress* module in Stata statistical software version 14 (StataCorp) was used to perform a fixed effects linear regression of effect size against SE with inverse variance weighting. The regression model included SE of ln(DOR), Modality (represented by indicator variable; CT, MRI, PET, ultrasonography, and other) with CT as the reference modality, time from publication of primary study to year of meta-analysis publication, and an interaction term *Modality* × *SE of ln(DOR)*. The interaction term assesses whether the slope coefficient for the SE of ln(DOR) is the same across the different modalities compared with the reference modality. The regression used robust SEs with clustering by meta-analysis. *P* values were 2-sided, and statistical significance was set at *P* = .05. Data were analyzed from August 24, 2021, to July 11, 2022.

## Results

### Search Results

**Figure 1.**
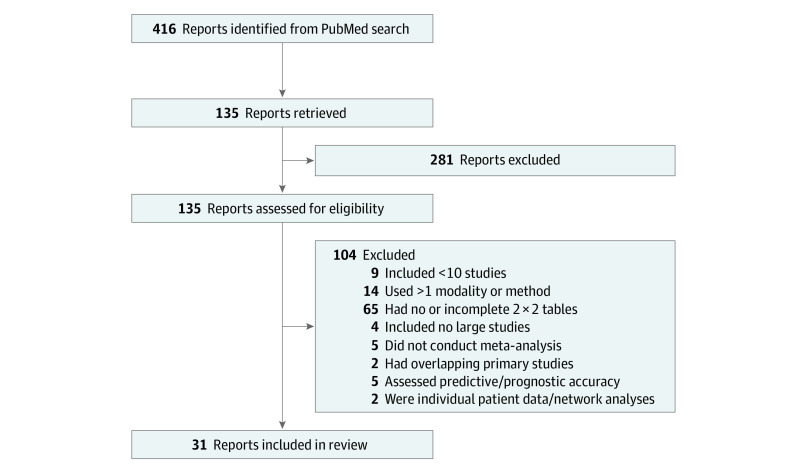
Study Assessment and Inclusion Flowchart

The search strategy is presented in Figure 1. The search identified 416 results. Of these, 281 were excluded during screening of titles and abstracts, and the remaining 135 potentially eligible articles were retrieved for full-text review. A total of 31 meta-analyses^25,26,27,28,29,30,31,32,33,34,35,36,37,38,39,40,41,42,43,44,45,46,47,48,49,50,51,52,53,54,55^ were included, with a total of 668 primary studies assessing 80 206 patients. Of these primary studies, the smallest trial included 5 participants; the largest, 3200 participants; there was a mean (SD) of 122 (186) participants and median (IQR) of 80 (46-137) participants per study. A total of 34 357 participants with disease and 45 949 participants without disease were included.

### Study Characteristics

Characteristics of included meta-analyses are shown in Table 1 and the eTable in the Supplement. The most commonly investigated imaging modalities were MRI (11 studies^25,26,28,32,33,41,46,48,51,52,55^), ultrasonography (8 studies^30,31,35,38,42,43,49,50^), CT (5 studies^29,37,44,45,47^), and PET (2 studies^27,54^). The target conditions included breast lesions, adrenal masses, and coronary artery disease. The earliest meta-analysis^55^ included was published in 2010, the latest^48^ in 2019.

**Table 1.** Summary of Included Meta-Analyses

Characteristic	No. (%)	
	Meta-analyses (n = 31)	Primary studies (n = 668)
Year of publication		
<2016	13 (41.9)	NA
≥2016	18 (58.1)	NA
Modality assessed		
CT	5 (16.1)	156 (23.4)
MRI	11 (35.5)	190 (28.4)
Ultrasonography	8 (25.8)	44 (6.6)
Nuclear medicine	2 (6.6)	206 (30.8)
Other	5 (16.1)	72 (10.8)

Abbreviations: CT, computed tomography; MRI, magnetic resonance imaging; NA, not applicable.

### Statistical Analysis

**Figure 2.**
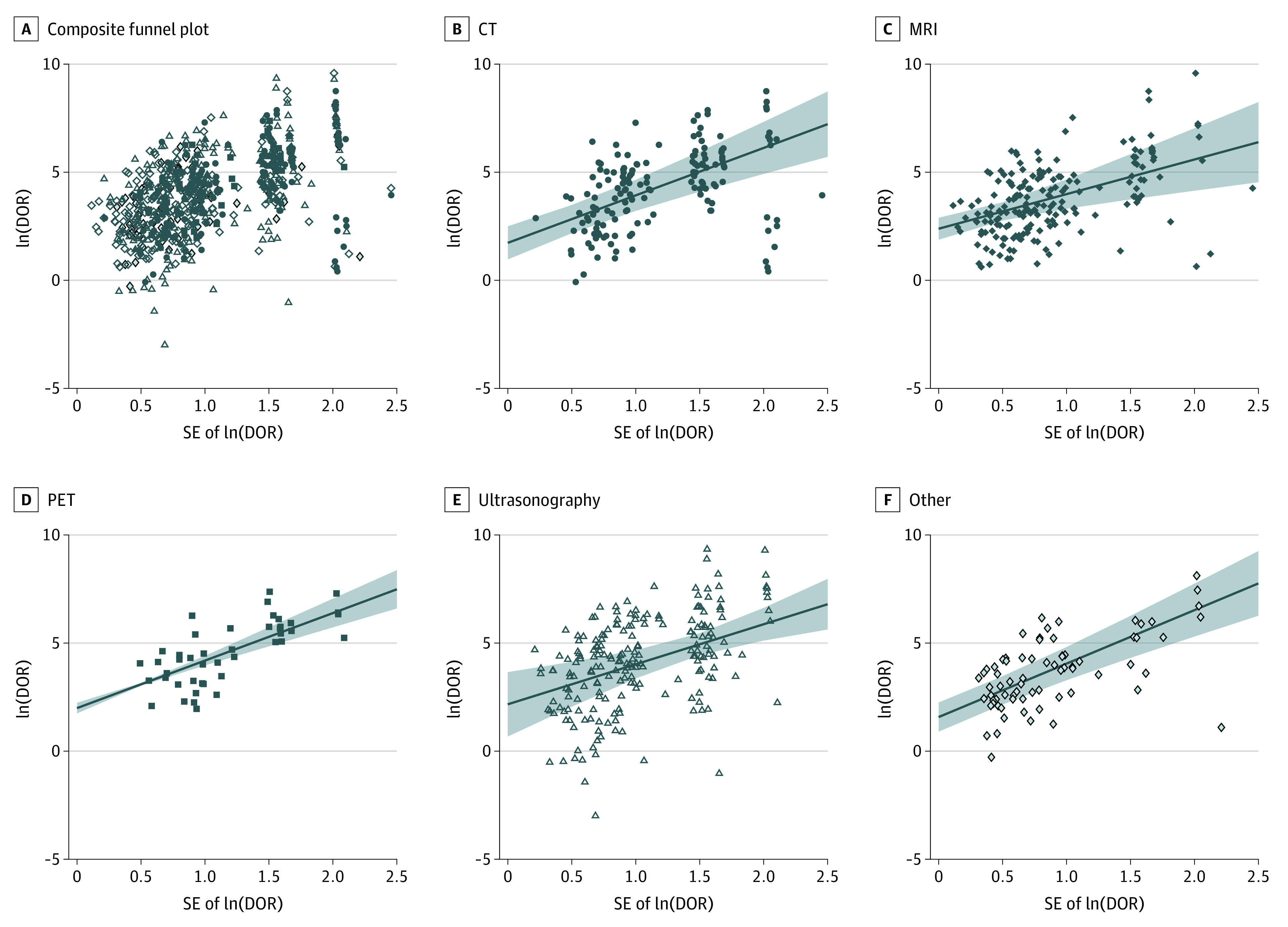
Composite Funnel Plots of the Associations Between Natural Log of the Diagnostic Odds Ratio (ln[DOR]) and SE of ln(DOR) Each marker represents a primary diagnostic accuracy study from one of the 31 included meta-analyses. Solid lines indicate the predictive margins; shading, 95% CIs; CT, computed tomography; MRI, magnetic resonance imaging; PET, positron emission tomography.

Figure 2 shows the composite funnel plot of ln(DOR) against SE. On visual observation, the plot is skewed such that there was a relative lack of studies that had higher SE with a small ln(DOR). Visual analysis of individual modalities revealed similar trends (Figures 2B-F). Fixed effects analysis produced a regression coefficient of ln(DOR) against SE of ln(DOR) of 2.19 (95% CI, 1.49-2.90; *P* < .001), with CT as the reference modality. The interaction term including other modalities did not demonstrate significantly different slope coefficients, thus providing evidence for an inverse association between effect size and precision across all primary studies, independent of modality (Table 2).

**Table 2.** Estimates of Regression Fixed Effects

Factor	Coefficient (95% CI)	Wald	*df*	*P* value
Constant	2.16 (1.35 to 2.97)	29.77	1	<.001
SE of ln(DOR)	2.19 (1.49 to 2.90)	40.4	1	<.001
Years since publication	–0.06 (–0.11 to –0.004)	4.89	1	.04
Fixed effects, Modality^a^		1.52	4	.22
MRI	0.49 (–0.44 to 1.41)	1.16	1	.29
PET	0.22 (–0.56 to 1.00)	0.34	1	.57
Ultrasonography	0.81 (–0.87 to 2.50)	0.98	1	.33
Other	–0.39 (–1.43 to 0.64)	0.60	1	.44
Interaction, Modality × SE of ln(DOR)		0.86	4	.50
MRI	–0.59 (–1.68 to 0.49)	1.24	1	.27
PET	0.003 (–0.81 to 0.82)	0.00	1	.99
Ultrasonography	–0.34 (–1.55 to 0.86)	0.34	1	.57
Other	0.28 (–0.64 to 1.19)	0.38	1	.54

Abbreviations: ln(DOR), natural log of the diagnostic odds ratio; MRI, magnetic resonance imaging; PET, positron emission tomography.

^a^ The reference modality was computed tomography.

The earliest primary study was published in 1981, and the latest in 2018. The years from publication of primary study to year of publication of the meta-analyses was also included in the fixed effects model, with a coefficient of −0.06 (95% CI, −0.11 to −0.004; *P* = .04), demonstrating a dependent association between time from publication and effect size. That is, more recently published studies were more likely to report higher DOR.

### Assessment of Publication Bias by Meta-Analyses

Of 31 included meta-analyses, all discussed publication bias to some extent (Table 3). Of these, 5 studies^41,42,43,48,55^ (16.1%) did not conduct a formal assessment. The common reason provided was the lack of evidence that methods used for assessment of publication bias in interventional studies would provide accurate results when used for diagnostic imaging accuracy research. Of 26 meta-analyses that included a formal assessment of publication bias,^25,26,27,28,29,30,31,32,33,34,35,36,37,38,39,40,44,45,46,47,49,50,51,52,53,54,55^ almost all (23 studies [88.5%]) used a visual assessment of funnel plots in addition to statistical tests. Most studies (21 studies [80.8%]) found no evidence for publication bias. Of the 4 studies that found publication bias, 2 studies used methods to adjust for the bias, while the remaining 2 studies did not.

**Table 3.** Assessment of Publication Bias by Meta-Analyses

Parameter	Studies, No. (%) (N = 31)
Mentioned publication bias	
Did not conduct formal assessment	5 (16.1)
Conducted formal assessment	26 (83.9)
Results of formal assessment	
No mention of result	1 (3.8)
Nil evidence for publication bias	21 (80.8)
Found evidence of publication bias	
Nil adjustment for publication bias	2 (7.7)
Adjusted for publication bias	2 (7.7)
Methods used in formal assessment	
Purely visual assessment of funnel plots	2 (7.7)
Purely statistical	1 (3.8)
Funnel plots and statistical tests	23 (88.5)

## Discussion

This meta-analysis assessed small study effects, their prevalence, and their effects in the medical imaging literature. Our assessment of 668 primary studies from 31 diagnostic imaging accuracy meta-analyses found an association between study precision and reported diagnostic accuracy, in which there was a tendency for studies of smaller precision to report a higher DOR. Since studies with lower precision generally represent those with smaller sample size, these results are compatible with small study effects. The observation was consistent across individual modalities (MRI, CT, PET, and ultrasonography). Taken together, these results indicate that small study effects are widely present throughout the diagnostic imaging literature.

Our findings of small study effects are consistent with those from a 2002 study by Song et al^16^ of the diagnostic test accuracy meta-analyses, which found that studies with smaller sample size tended to report better test performance. The study by Song et al^16^ included all diagnostic tests, both imaging and laboratory tests, and did not conduct a subgroup analysis to assess for differences between these types of diagnostic tests. However, our findings contrasted with those from another meta-epidemiological study by van Enst et al^17^ that found that studies with larger sample size reported higher accuracy. Key differences between the study by van Enst et al^17^ and our study include the inclusion of studies of all diagnostic tests vs our examination of diagnostic imaging studies only, the use of sample size in regression analysis vs SE of ln(DOR), and the use of the Firths method^56^ to correct for zero values in their calculations of DOR vs our use of the Haldane-Anscombe method^22,23^ of adding 0.5 to all cells. In particular, the use of sample size in the funnel plot may lead to unpredictable results,^1^ while SE is preferable, as it summarizes all factors that influence statistical power.^57^

There are several possible explanations for the observed small study effects. Publication bias exists broadly throughout the biomedical literature^58^ and is a key contributor to small study effects.^2^ For example, in the medical imaging literature, it has been demonstrated that a positive study outcome in abstracts presented at radiological meetings is associated with subsequent publication in a peer-reviewed journal.^59,60^ It is generally accepted that larger studies are more rigorous and well controlled and less prone to publication bias for several reasons, including the likelihood of being preregistered, the involvement of more experienced research teams, and the time and money invested.^2,61,62^ In contrast, smaller studies require less time and resources to conduct, which may be important when investigating novel technologies with an exploratory purpose but can be more prone to bias.^63^

Apart from publication bias, other potential explanations for small study effects have been identified.^9^ These include other forms of reporting bias, such as selective outcome reporting and selective analysis reporting, true heterogeneity, or the presence of low-quality small studies producing greatly inflated effect sizes.^64^ True heterogeneity can contribute to small study effects when study characteristics are related to the size of the study, such as different types of participants or intensity of interventions.^2,65^ This may arise in the selection of more expensive and less frequently used imaging modalities that have higher diagnostic accuracy only for select patients, such as catheter angiography vs CT or MRI for investigation of vascular pathologies.^66^ Thus, while publication bias is likely a key cause of small study effects, it is difficult to ascertain the relative influence of other factors in our study.

Funnel plot asymmetry was not commonly found in diagnostic imaging studies when assessed at the level of individual meta-analyses by the original authors. One explanation is that meta-analyses often may not have sufficient sample size nor power for the frequently used methods of assessing for small study effects to detect such effects.^2,57^ We therefore excluded meta-analyses with fewer than 10 primary studies. However, this minimum number of studies to produce an accurate estimate of funnel plot asymmetry depends on a variety of factors.^57^ A simulation study found that the Egger, Macaskill, and Begg tests had either very low power or were misleading owing to heterogeneous diagnostic thresholds not present in the interventional research for which these tests were designed.^21^ An evaluation of 6873 meta-analyses found that only 366 (5%) met the statistical conditions required for the appropriate use and interpretation of funnel plot asymmetry tests.^67^ These studies, taken together with our findings, indicate that the presence of small study effects, and possibly publication bias, may be underdetected and underestimated, even when formally assessed in meta-analyses of diagnostic imaging accuracy.

Diagnostic imaging research informs the selection, use, and interpretation of imaging tests. The notion that small study effects and publication bias may be widely present yet difficult to detect has the potential to undermine many meta-analyses in diagnostic imaging. The potential of studies to overestimate the diagnostic accuracy of a test owing to this has implications for cost, utilization of imaging, and even patient outcomes by suboptimal choice of imaging test or unnecessary exposure to ionizing radiation. To mitigate some of these issues, we suggest that readers and authors be aware that commonly used tests for funnel plot asymmetry often do not have enough power in diagnostic imaging meta-analyses and thus may underestimate or fail to detect the presence of underlying bias.^16^ Emphasizing the size and direction of funnel plot asymmetry may be more appropriate for diagnostic test meta-analyses, as opposed to using a threshold for statistical significance.^16^ Further research is required to explore the factors that contribute to small study effects (eg, publication bias and heterogeneity), quantify their impact on pooled results at the meta-analysis level, and identify appropriate methods to adequately adjust for their impact.

### Limitations

Our study has some limitations. We chose to exclude meta-analyses that included fewer than 10 primary studies (9 were excluded for this reason) and those that did not provide 2 × 2 contingency data (65 were excluded for this reason), resulting in a sample of 31 meta-analyses.^25,26,27,28,29,30,31,32,33,34,35,36,37,38,39,40,41,42,43,44,45,46,47,48,49,50,51,52,53,54,55^ Despite this sample size, our results still showed significant evidence for small study effects. Additionally, as zero values were present in the 2 × 2 contingency data, we added 0.5 to all values to enable calculation of DOR.^61,68^ This zero correction method is an approximately unbiased estimator of the true OR^62,69^ and affects smaller values for DOR more than larger. Consequently, the correction may actually skew the calculation of DOR such that small study effects are actually underestimated.^70^ It is difficult to ascertain the extent to which different factors contribute to the small study effects observed in this study.

## Conclusions

This meta-analysis of diagnostic imaging accuracy meta-analyses found that small study effects were widely present throughout the literature and likely underestimated at the level of individual meta-analyses. These findings have significant implications on the conduct and interpretation of meta-analyses in the diagnostic imaging literature, as they suggest that diagnostic accuracy estimates presented by many meta-analyses may be gross overestimates.

## Notes

Supplement. **eAppendix.** Search Strategy **eTable.** Summary of Included Meta-analysis
eReferences.
