# Supplementary material for: Small Study Effects in Diagnostic Imaging Accuracy: A Meta-Analysis
Source: JAMA Netw Open. 2022 Aug 25;5(8):e2228776. doi: 10.1001/jamanetworkopen.2022.28776 (PMC9412222; doi:10.1001/jamanetworkopen.2022.28776)
Supplement: Supplement. — eAppendix. Search Strategy eTable. Summary of Included Meta-analysis eReferences. [file jamanetwopen-e2228776-s001.pdf]

# Supplemental Online Content

Lu L, Phua QS, Bacchi S, et al. Small study effects in diagnostic imaging accuracy: a meta-analysis. *JAMA Netw Open*. 2022;5(8):e2228776.
doi:10.1001/jamanetworkopen.2022.28776

**eAppendix.** Search Strategy
**eTable.** Summary of Included Meta-analysis
**eReferences.**

This supplemental material has been provided by the authors to give readers additional information about their work.

© 2022 Lu L et al. *JAMA Network Open.*

**eAppendix.** Search Strategy

The search string used is as follows: "((meta-analysis[Title/Abstract]) OR (meta-analysis as topic[MeSH Terms]) OR(meta-analysis[Publication Type])) AND ((JACC-Cardiovascular Imaging[Journal]) OR (MEDICAL IMAGE ANALYSIS[Journal]) OR (RADIOLOGY[Journal]) OR (JOURNAL OF NUCLEAR MEDICINE[Journal]) OR (EUROPEAN JOURNAL OF NUCLEAR MEDICINE AND MOLECULAR IMAGING[Journal]) OR (IEEE TRANSACTIONS ON MEDICAL IMAGING[Journal]) OR (CLINICAL NUCLEAR MEDICINE[Journal]) OR (NEUROIMAGE[Journal]) OR (Photoacoustics[Journal]) OR (INTERNATIONAL JOURNAL OF RADIATION ONCOLOGY BIOLOGY PHYSICS[Journal]) OR (Circulation-Cardiovascular Imaging[Journal]) OR (Ultrasound Obstet Gynecol[Journal]) OR (JOURNAL OF CARDIOVASCULAR MAGNETIC RESONANCE[Journal]) OR (INVESTIGATIVE RADIOLOGY[Journal]) OR (RADIOGRAPHICS[Journal]) OR (ULTRASCHALL IN DER MEDIZIN[Journal]) OR (RADIOTHERAPY AND ONCOLOGY[Journal]) OR (European Heart Journal-Cardiovascular Imaging[Journal]) OR (HUMAN BRAIN MAPPING[Journal]) OR (Journal of the American College of Radiology[Journal]) OR (EUROPEAN RADIOLOGY[Journal]) OR (SEMINARS IN RADIATION ONCOLOGY[Journal]) OR (JOURNAL OF MAGNETIC RESONANCE IMAGING[Journal]) OR (Biomedical Optics Express[Journal]) OR (Comput Med Imaging Graph[Journal]) OR (JOURNAL OF DIGITAL IMAGING[Journal]) OR (MAGNETIC RESONANCE IN MEDICINE[Journal]) OR (Insights into Imaging[Journal]) OR (INTERNATIONAL JOURNAL OF HYPERTHERMIA[Journal]) OR (SEMINARS IN NUCLEAR MEDICINE[Journal])) AND ((diagnostic test accuracy OR DTA [all fields]) OR (sensitivity and specificity [all fields]) OR (sensitiv*[all fields]) OR(specificit*[all fields]) OR (accuracy[all fields]) OR (ROC [all fields]) OR ("receiver operator characteristic" [all fields]) OR ("diagnostic odds ratio" [all fields]) OR ("likelihood ratio" [all fields]) OR ("positive predictive value" [all fields]) OR (PPV [all fields]) OR ("negative predictive value"[all fields]) OR (NPV[all fields]) OR (false positive*[all fields]) OR (false negative*[all fields])) AND (2010/01/01[PDat]:2019/12/31[PDat])".

© 2022 Lu L et al. *JAMA Network Open.*

**eTable.** Summary of Included Meta-analysis

| First author name | Topic | Investigation | Number of studies | Number of subjects |
|---|---|---|---|---|
| Baltzer, P. A. et al.(1) 2013 | Proton MR spectroscopy for breast lesions | Proton MR spectroscopy | 19 | 1200 |
| Bennani-Baiti, B.(2) 2017 | MRI for mammographic microcalcifications | MRI | 20 | 1843 |
| Boland, G.(3) 2011 | FDG PET for adrenal masses | FDG PET | 21 | 1390 |
| Chen, L.(4) 2013 | dwMRI for lung lesions | dwMRI | 11 | 1035 |
| Dai, T.(5) 2018 | CTA for coronary artery disease | CTA | 34 | 3926 |
| Dong, J.(6) 2014 | Ultrasound for Crohn's disease | Ultrasound | 15 | 1588 |
| Guerriero, S.(7) 2016 | Transvaginal ultrasound for endometriosis in the rectosigmoid | Transvaginal ultrasound | 19 | 2639 |
| Jie, C.(8) 2014 | Diffusion-weighted imaging for prostate cancer | Diffusion-weighted imaging | 21 | 11520 |
| Kierans, A. S.(9) 2016 | Dynamic contrast-enhanced MRI for hepatocellular carcinoma | Dynamic contrast-enhanced MRI | 22 | 2368 |
| Lin, P.(10) 2014 | Shear wave elastography for malignant thyroid nodules | Shear wave elastography | 15 | 1849 |
| Ma, X.(11) 2015 | Contrast-enhanced ultrasound for ovarian tumours | Contrast-enhanced ultrasound | 11 | 817 |
| Meng, Z.(12) 2015 | Automated breast volume scanner for malignant and benign breast lesions | Automated breast volume scanner | 13 | 1774 |
| Menke, J.(13) 2016 | Coronary CTA for coronary artery disease | Coronary CTA | 30 | 3041 |
| Mojadidi, M. K.(14) 2014 | Transcranial Doppler for intracardiac right-to-left shunt | Transcranial Doppler | 29 | 1897 |
| Nudi, F.(15) 2017 | Myocardial perfusion imaging for coronary artery disease | Myocardial perfusion imaging | 16 | 2092 |
| Platzek, I.(16) 2019 | Chemical shift imaging for adrenal masses | Chemical shift imaging | 18 | 1281 |

© 2022 Lu L et al. *JAMA Network Open.*

| | | | | |
|---|---|---|---|---|
| Repplinger, M. D.[17] 2016 | MRI for appendicitis | MRI | 10 | 890 |
| Richardson, A.[18] 2015 | Ultrasound for intrauterine pregnancy | Ultrasound | 22 | 3008 |
| Richardson, A.[19] 2016 | Ultrasound for tubal ectopic pregnancy | Ultrasound | 42 | 7124 |
| Romero, J(20) 2013 | Cardiac CT for left atrial appendage thrombus in patients with atrial fibrillation | Cardiac CT | 19 | 2955 |
| Sevcenco, S.[21] 2017 | CT for renal cystic lesions | CT | 35 | 2504 |
| Shakoor, D.[22] 2019 | 3D MRI for knee meniscal injuries | 3D MRI | 31 | 4134 |
| Somers, I.[23] 2017 | Contrast-enhanced CT for pancreatic carcinoma | Contrast-enhanced CT | 38 | 2494 |
| Wade, R. G.[24] 2019 | MRI for root avulsions in traumatic adult brachial plexus injuries | MRI | 14 | 1154 |
| Wang, Q.[25] 2016 | MR spectroscopy for gliomas | MR spectroscopy | 11 | 432 |
| Wu, M.[26] 2018 | Contrast-enhanced ultrasound for focal liver lesions | Contrast-enhanced ultrasound | 57 | 9338 |
| Xia, D.[27] 2010 | drMRI for focal hepatic lesions | drMRI | 14 | 1675 |
| Zhang, L.[28] 2017 | MRI for prostate cancer | MRI | 14 | 2411 |
| Zhang, Y.[29] 2019 | Sonoelastography for parotid lesions | Sonoelastography | 10 | 725 |
| Zhou, H.[30] 2016 | FDG PET/CT for nasopharyngeal carcinoma | FDG PET/CT | 23 | 1262 |
| Zijta, F.[31] 2010 | MR colonography for colorectal lesions | MR colonography | 14 | 1322 |

CT, computed tomography; CTA, computed tomography angiography; dwMRI, diffusion-weighted magnetic resonance imaging; FDG, fluorodeoxyglucose; MR, magnetic resonance; MRI, magnetic resonance imaging; PET, positron emission tomography.

© 2022 Lu L et al. *JAMA Network Open.*

**eReferences**


1. Baltzer PA, Dietzel M. Breast lesions: diagnosis by using proton MR spectroscopy at 1.5 and 3.0 T--systematic review and meta-analysis. Radiology. 2013;267(3):735-46.
2. Bennani-Baiti B, Baltzer PA. MR Imaging for Diagnosis of Malignancy in Mammographic Microcalcifications: A Systematic Review and Meta-Analysis. Radiology. 2017;283(3):692-701.
3. Boland GW, Dwamena BA, Jagtiani Sangwaiya M, Goehler AG, Blake MA, Hahn PF, et al. Characterization of adrenal masses by using FDG PET: a systematic review and meta-analysis of diagnostic test performance. Radiology. 2011;259(1):117-26.
4. Chen L, Zhang J, Bao J, Zhang L, Hu X, Xia Y, et al. Meta-analysis of diffusion-weighted MRI in the differential diagnosis of lung lesions. J Magn Reson Imaging. 2013;37(6):1351-8.
5. Dai T, Wang JR, Hu PF. Diagnostic performance of computed tomography angiography in the detection of coronary artery in-stent restenosis: evidence from an updated meta-analysis. Eur Radiol. 2018;28(4):1373-82.
6. Dong J, Wang H, Zhao J, Zhu W, Zhang L, Gong J, et al. Ultrasound as a diagnostic tool in detecting active Crohn's disease: a meta-analysis of prospective studies. Eur Radiol. 2014;24(1):26-33.
7. Guerriero S, Ajossa S, Orozco R, Perniciano M, Jurado M, Melis GB, et al. Accuracy of transvaginal ultrasound for diagnosis of deep endometriosis in the rectosigmoid: systematic review and meta-analysis. Ultrasound Obstet Gynecol. 2016;47(3):281-9.
8. Jie C, Rongbo L, Ping T. The value of diffusion-weighted imaging in the detection of prostate cancer: a meta-analysis. Eur Radiol. 2014;24(8):1929-41.
9. Kierans AS, Kang SK, Rosenkrantz AB. The Diagnostic Performance of Dynamic Contrast-enhanced MR Imaging for Detection of Small Hepatocellular Carcinoma Measuring Up to 2 cm: A Meta-Analysis. Radiology. 2016;278(1):82-94.
10. Lin P, Chen M, Liu B, Wang S, Li X. Diagnostic performance of shear wave elastography in the identification of malignant thyroid nodules: a meta-analysis. Eur Radiol. 2014;24(11):2729-38.
11. Ma X, Zhao Y, Zhang B, Ling W, Zhuo H, Jia H, et al. Contrast-enhanced ultrasound for differential diagnosis of malignant and benign ovarian tumors: systematic review and meta-analysis. Ultrasound Obstet Gynecol. 2015;46(3):277-83.
12. Meng Z, Chen C, Zhu Y, Zhang S, Wei C, Hu B, et al. Diagnostic performance of the automated breast volume scanner: a systematic review of inter-rater reliability/agreement and meta-analysis of diagnostic accuracy for differentiating benign and malignant breast lesions. Eur Radiol. 2015;25(12):3638-47.
13. Menke J, Kowalski J. Diagnostic accuracy and utility of coronary CT angiography with consideration of unevaluable results: A systematic review and multivariate Bayesian random-effects meta-analysis with intention to diagnose. Eur Radiol. 2016;26(2):451-8.
14. Mojadidi MK, Roberts SC, Winoker JS, Romero J, Goodman-Meza D, Gevorgyan R, et al. Accuracy of transcranial Doppler for the diagnosis of intracardiac right-to-left shunt: a bivariate meta-analysis of prospective studies. JACC Cardiovasc Imaging. 2014;7(3):236-50.
15. Nudi F, Iskandrian AE, Schillaci O, Peruzzi M, Frati G, Biondi-Zoccai G. Diagnostic Accuracy of Myocardial Perfusion Imaging With CZT Technology: Systemic Review and Meta-Analysis of Comparison With Invasive Coronary Angiography. JACC Cardiovasc Imaging. 2017;10(7):787-94.


© 2022 Lu L et al. *JAMA Network Open.*

16. Platzek I, Sieron D, Plodeck V, Borkowetz A, Laniado M, Hoffmann RT. Chemical shift imaging for evaluation of adrenal masses: a systematic review and meta-analysis. Eur Radiol. 2019;29(2):806-17.
17. Repplinger MD, Levy JF, Peethumnongsin E, Gussick ME, Svenson JE, Golden SK, et al. Systematic review and meta-analysis of the accuracy of MRI to diagnose appendicitis in the general population. J Magn Reson Imaging. 2016;43(6):1346-54.
18. Richardson A, Gallos I, Dobson S, Campbell BK, Coomarasamy A, Raine-Fenning N. Accuracy of first-trimester ultrasound in diagnosis of intrauterine pregnancy prior to visualization of the yolk sac: a systematic review and meta-analysis. Ultrasound Obstet Gynecol. 2015;46(2):142-9.
19. Richardson A, Gallos I, Dobson S, Campbell BK, Coomarasamy A, Raine-Fenning N. Accuracy of first-trimester ultrasound in diagnosis of tubal ectopic pregnancy in the absence of an obvious extrauterine embryo: systematic review and meta-analysis. Ultrasound Obstet Gynecol. 2016;47(1):28-37.
20. Romero J, Husain SA, Kelesidis I, Sanz J, Medina HM, Garcia MJ. Detection of left atrial appendage thrombus by cardiac computed tomography in patients with atrial fibrillation: a meta-analysis. Circ Cardiovasc Imaging. 2013;6(2):185-94.
21. Sevcenco S, Spick C, Helbich TH, Heinz G, Shariat SF, Klingler HC, et al. Malignancy rates and diagnostic performance of the Bosniak classification for the diagnosis of cystic renal lesions in computed tomography - a systematic review and meta-analysis. Eur Radiol. 2017;27(6):2239-47.
22. Shakoor D, Kijowski R, Guermazi A, Fritz J, Roemer FW, Jalali-Farahani S, et al. Diagnosis of Knee Meniscal Injuries by Using Three-dimensional MRI: A Systematic Review and Meta-Analysis of Diagnostic Performance. Radiology. 2019;290(2):435-45.
23. Somers I, Bipat S. Contrast-enhanced CT in determining resectability in patients with pancreatic carcinoma: a meta-analysis of the positive predictive values of CT. Eur Radiol. 2017;27(8):3408-35.
24. Wade RG, Takwoingi Y, Wormald JCR, Ridgway JP, Tanner S, Rankine JJ, et al. MRI for Detecting Root Avulsions in Traumatic Adult Brachial Plexus Injuries: A Systematic Review and Meta-Analysis of Diagnostic Accuracy. Radiology. 2019;293(1):125-33.
25. Wang Q, Zhang H, Zhang J, Wu C, Zhu W, Li F, et al. The diagnostic performance of magnetic resonance spectroscopy in differentiating high-from low-grade gliomas: A systematic review and meta-analysis. Eur Radiol. 2016;26(8):2670-84.
26. Wu M, Li L, Wang J, Zhang Y, Guo Q, Li X, et al. Contrast-enhanced US for characterization of focal liver lesions: a comprehensive meta-analysis. Eur Radiol. 2018;28(5):2077-88.
27. Xia D, Jing J, Shen H, Wu J. Value of diffusion-weighted magnetic resonance images for discrimination of focal benign and malignant hepatic lesions: a meta-analysis. J Magn Reson Imaging. 2010;32(1):130-7.
28. Zhang L, Tang M, Chen S, Lei X, Zhang X, Huan Y. A meta-analysis of use of Prostate Imaging Reporting and Data System Version 2 (PI-RADS V2) with multiparametric MR imaging for the detection of prostate cancer. Eur Radiol. 2017;27(12):5204-14.
29. Zhang YF, Li H, Wang XM, Cai YF. Sonoelastography for differential diagnosis between malignant and benign parotid lesions: a meta-analysis. Eur Radiol. 2019;29(2):725-35.
30. Zhou H, Shen G, Zhang W, Cai H, Zhou Y, Li L. 18F-FDG PET/CT for the Diagnosis of Residual or Recurrent Nasopharyngeal Carcinoma After Radiotherapy: A Metaanalysis. J Nucl Med. 2016;57(3):342-7.
31. Zijta FM, Bipat S, Stoker J. Magnetic resonance (MR) colonography in the detection of colorectal lesions: a systematic review of prospective studies. Eur Radiol. 2010;20(5):1031-46.

© 2022 Lu L et al. *JAMA Network Open.*
